# Isolation and characterization of *Babesia pecorum* sp. nov. from farmed red deer (*Cervus elaphus*)

**DOI:** 10.1186/s13567-014-0078-7

**Published:** 2014-08-26

**Authors:** Maggy Jouglin, Isabel G Fernández-de-Mera, Nathalie de la Cotte, Francisco Ruiz-Fons, Christian Gortázar, Emmanuelle Moreau, Suzanne Bastian, José de la Fuente, Laurence Malandrin

**Affiliations:** INRA, UMR1300 BioEpAR, F-44307 Nantes, France; LUNAM Université, Oniris, Ecole nationale vétérinaire, agroalimentaire et de l’alimentation Nantes-Atlantique, UMR BioEpAR, F-44307 Nantes, France; Health & Biotechnology (SaBio) Group, IREC (CSIC-UCLM- JCCM), Ronda de Toledo s/n, 13005 Ciudad Real, Spain; Centro de Vigilancia Sanitaria (VISAVET). Ave. Puerta de Hierro s/n, Universidad Complutense de Madrid, Madrid, Spain; Department of Veterinary Pathobiology, Center for Veterinary Health Sciences, Oklahoma State University, Stillwater, OK 74078 USA

## Abstract

The diversity of *Babesia* species infecting cervids in parts of central and southern Spain was analyzed by collecting blood from farmed red deer (*Cervus elaphus*). *Babesia* sp. was isolated in vitro from two red deer herds in Cádiz and Ciudad Real. The number of *Babesia* sp. carriers differed between the two herds: 36/77 in Cádiz and 1/35 in Ciudad Real. *Hyalomma lusitanicum* was the most prevalent tick species identified on the Cádiz farm vegetation and on sampled animals, and is therefore a candidate vector. The molecular characteristics of 21 isolates were determined by complete (8 isolates) or partial (13 isolates) 18S rRNA gene sequencing. The sequences were highly similar (over 99.4% identity) and 6 sequence types were identified at the level of one herd only, demonstrating a rather high genetic diversity. They formed a monophyletic clade, and members of the three main sequence types shared a similar morphology and the same erythrocyte susceptibility pattern. This clade also included *Babesia* sp. Xinjiang isolated from sheep in China and *Babesia* sp. identified in giraffe in South Africa, with identities higher than 98.3% and statistically relevant phylogenetic support. None of the biological properties analyzed for both *Babesia* from red deer and *Babesia* sp. Xinjiang allowed their differentiation (ability to develop in vitro in erythrocytes from cattle and sheep, as well as in erythrocytes from different cervids, unsuccessful infection of calves). We propose the *Babesia* isolated from red deer as a new species named *B. pecorum.* Whether *Babesia* sp. Xinjiang and the *Babesia* characterized in South Africa belong to the same species is debated.

**Electronic supplementary material:**

The online version of this article (doi:10.1186/s13567-014-0078-7) contains supplementary material, which is available to authorized users.

## Introduction

Piroplasms of the genus *Babesia* are tick-borne parasitic protozoa responsible for infections referred to as babesiosis. Some species of *Babesia* are pathogenic for domestic ruminants (cattle, sheep and goats) and/or wild ruminants (cervids). The latter have been proven or suspected to act as reservoirs for domestic hosts [1–3]. Ruminants are also reservoirs of zoonotic *Babesia* such as *B. divergens* (cattle) and *Babesia* sp. EU1 (roe deer, *Capreolus capreolus*) [4,5]. In recent decades, wildlife farming - especially deer farming − has expanded worldwide. This expansion is a reflex of the demand of venison and live individuals for restocking purposes in hunting activities [6], and of antler velvet for Asian traditional medicine markets [7]. However, infectious diseases of these wildlife ungulates are not well characterized [8], and may pose serious problems in the context of farming, with dense populations of individuals.

*B. capreoli*, *Babesia* sp. EU1 and potentially *B. divergens* have been reported in wild cervids in many European countries, but mortality caused by these agents is difficult to assess in wild animals [8–12]. Up to now, 18S rRNA has remained the reference gene for the identification and classification of organisms, and has been widely used for the molecular phylogenetic classification of piroplasms. Classifications based solely on molecular criteria do not, however, take into account the host range of the classified parasites, which must be known in order to determine host reservoirs and understand host-pathogen co-evolution. To access this biological data, the parasites need to be isolated which is an essential but labor intensive step.

The objective of the present study was to isolate and then identify, on the basis of their 18S rRNA gene sequences, *Babesia* species from farmed red deer (*Cervus elaphus*) in central and southern Spain. Ticks were also collected from the pastures and on animals, and identified in order to evaluate the range of possible vectors for the isolated *Babesia* species. Some biological features of the parasites isolated in vitro were then determined: i.e. morphology, host range analysis using in vitro erythrocyte susceptibility tests and experimental infections. The description of the parasites isolated from red deer as a new species is proposed based on the collected data.

## Materials and methods

### Blood sampling

Blood samples were collected from 112 red deer (77 from a farm in Cádiz and 35 from a farm in Ciudad Real) from December 2010 to February 2011 (Table 1). Peripheral blood was aseptically punctured into 5 mL evacuated glass tubes containing heparin as an anticoagulant. The time between sample collection and processing varied from 2 to 7 days. Blood samples were stored at 4 °C before in vitro culture initiation.

**Table 1:** **Description of red deer blood samples, of the number of collected isolates and 18S rRNA gene sequences obtained**

	**Location**		
	**Cádiz**	**Ciudad Real**	**Total**
Number of samples	77	35	112
Number of isolates	36	1	37
Number of complete 18S rRNA sequences (>1616 bp)	7	1	8
Number of partial 18S rRNA sequences	13	0	13

The presence of hemoparasites in the collected samples was checked by light microscopy of thin May-Grünwald-Giemsa (MGG)-stained blood smears. At least 30 microscope fields (about 10 000 erythrocytes) were examined.

### In vitro isolation of *Babesia* spp.

Hemoparasites were isolated from red deer blood by preparing in vitro cultures of *Babesia* spp. from each blood sample with the method described previously [13]. Cultures were prepared in autologous red blood cells as well as in bovine red blood cells from a blood donor. The cow used as a blood donor was selected in an experimental herd and kept indoors from birth in specific experimental facilities. The whole herd has been tested and proved to be free from *Babesia* and *Theileria*. Initial cultures were maintained for a period of 3 weeks, replacing the medium every two to three days. This period was shown to be necessary for the development of slow growing isolates [13]. Parasite presence was checked on Giemsa-stained smears 6, 15 and 21 days after culture initiation or when hemolysis was observed in culture wells.

### Molecular identification of *Babesia* isolated in vitro

Infected erythrocyte pellets from an in vitro culture were washed with phosphate buffer saline (PBS) and frozen at −20 °C in an equal volume of PBS. Genomic DNA (gDNA) was purified from parasitized RBC using the Promega Wizard gDNA purification kit (Promega, France). The obtained isolates were identified by PCR amplification of the 18S rRNA using primers CryptoF (5'-AACCTGGTTGATCCTGCCAGTAGTCAT-3') and CryptoR (5'-GAATGATCCTTCCGCAGGTTCACCTAC- 3') for the complete sequences and primers BabGF2 (5'-GYYTTGTAATTGGAATGATGG-3') and BabGR2 (5'-CCAAAGACTTTGATTTCTCTC-3') for the partial sequences covering the hypervariable region of the 18S rRNA (467–1026 bp). The amplicons in both strands were then directly sequenced (Sequencing Platform of BioGenOuest, Nantes, France) by using the internal primers for the full length sequence (Cryp-up2 5'-GCCGCCTAGGGATTGGAGG-34 and Cryp-down 5'-CTGCTGGCACCAGACTTGCC-3') [11] and assembled.

The 18S rRNA complete nucleotide sequence data generated for 4 isolates representing the observed sequence diversity were deposited in GenBank database [14] under accession numbers KC249943-KC249946. Two partial sequences that differ from these 4 sequences were deposited under GenBank accession numbers KC249947 and KC249948.

### Sequence analysis, alignment, and phylogenetic analysis

The sequences obtained were subjected to BLASTn identity searches in the GenBank database. Multiple sequence alignments were performed using the program Clustal W [15] to highlight differences between the red deer 18S rRNA sequences and to perform comparisons with related sequences found in the NCBI database [14].

Two sets of phylogenetic trees were constructed. The first was constructed to place the new red deer isolates in the *Babesia* clade and define a suitable outgroup for a more refined analysis. For this purpose, 20 sequences representing the main *Babesia* sub-clades [16,17] were selected and aligned with the sequence of the main representative of the red deer isolate sequences. The alignment was truncated manually to the size of the shortest sequence. Sequences of *Theileria annulata* and *Theileria buffeli* were used as outgroups, based on recently published phylogenetic analyses [16,17]. Gaps were automatically removed during the analyses by MEGA5. The final alignment consisted in 1391 bp.

A second set of alignments was performed using the 4 different complete red deer sequences as well as all the closely related sequences (5 other sequences from *Babesia* species isolated from Giraffe, sheep and roan antelope). *B. divergens* and *B. bigemina* 18S rDNA sequences were added as representative of other main sister clades. These sequences were also selected from the first set of alignment as they did not generate large indels, especially in the region where substitutions were described between the red deer and the closely related sequences. Following examination, the alignment was then manually optimized to remove indels. This optimization allowed the conservation of the 17 substitution positions described in the results section (Table 2). The alignment used for phylogenetic analysis contained 13 sequences and 1461 aligned positions.

**Table 2:** **Position and nature of the nucleotide changes among the sequences of the new** ***Babesia*** **isolated from red deer and closely related** ***Babesia*** **sp.**

**Isolate reference**	**Seq. type**	**Nucleotide position (DQ159073 as reference)**																					
		**186**	**216**	**223**	**224**	**247**	**469**	**494**	**623**	**628**	**638**	**639**	**645**	**662**	**663**	**774**	**792**	**801**	**830**	**975**	**1229**	**1438**	**1451**
262, 265, A2, A21	ST1	-	T	-	.	A	A	A	T	G	T	A	G	-	T	T	A	G	A	T	T	T	G
279, C32	ST2	.	.	.		.	T	.	.	.	.	.	.	.	.	.	.	.	.	.	.	.	.
A20	ST3	.	.	.		.	.	.	.	.	.	G	.	.	.	.	.	.	.	.	.	.	.
232	ST4	.	.	.		G	.	.	.	.	-	G	.	.	A	.	.	.	.	.	.	.	.
DQ159073 *B*. sp. Xinjiang		.	.	.		.	T	.	C	.	.	G	T	G	.	.	.	.	.	.	.	A	.
FJ213577 Giraffe 544		T	C	T		.	T	.	.	C	.	T	T	.	.	.	.	.	.	.	.	.	.
FJ213578 Giraffe 0105		T	C	T		.	T	.	.	C	.	C	T	.	.	C	G	.	.	C	C	.	.
FJ213580 Giraffe 229		T	C	T		.	T	G	.	C	.	T	T	.	.	.	.	A	G	.	.	.	A
FJ213581 Roan 571		T	C	T		.	T	G	.	C	.	T	T	.	.	.	.	A	.	.	.	.	.
156, 188, 312, A4, A39, A54, A55, A56, A65	ST1 or ST2								.	.	.	.	.	.	.	.	.	.	.				
A3, A64	ST5								.	.	.	.	T	.	.	.	.	.	.				
191, A66	ST6								.	.	.	G	.	.	A	.	.	.	.				

Complete sequences from this study are presented first and compared to complete sequences from the related *Babesia* (*Babesia* sp. Xinjiang from China and *Babesia* from giraffe and roan antelope in South Africa). The nucleotide position refers to the sequence DQ159073. Partial sequences from the *Babesia* isolated from red deer were compared between nucleotide positions 560 and 935. Dots (.) indicate sequence identity with the ST1 sequence. Dashes (−) indicate a deletion. Seq. type: sequence type.

The phylogenetic analyses were performed in MEGA5 [18] using the Neighbor-Joining, the Maximum Likelihood and the Maximum Parsimony methods. Parameters used for each method are indicated in the Additional files with the corresponding trees [19–21]. The Bayesian phylogenetic analysis was conducted using MrBayes v3.2.2 [22,23]. The GTR model of nucleotide substitution, with gamma-distributed frequencies and invariant sites, was used. The first analysis was run for 300 000 generations, the second with 400 000 generations and trees in both cases were sampled every 200 generations.

### Determination of in vitro erythrocyte susceptibility

In vitro erythrocyte susceptibility was determined for some isolates cultivated in vitro from red deer blood. Cultures in cattle, fallow deer and sheep erythrocytes (sheep 3264 and 15) were initiated with 5 × 10^5^ iRBC/mL of these parasites cultivated in bovine erythrocytes. Cultures were performed in 96-well U-bottom plates and growth was monitored by A_405_ of the culture supernatant as previously described [24]. Growth was measured at 5 time points between day 2 and day 5 after culture initiation. The ability to grow on roe deer erythrocytes was evaluated by simply subculturing the parasites from red deer erythrocytes into roe deer erythrocytes collected from a healthy (PCR tested) animal (Gardouch research station enclosure, INRA, CEFS).

### Experimental infection of calves

Two calves were infected with their own red blood cells which had been infected in vitro with isolate 265. This isolate was selected as representative of the main genetic group isolated in this study. One of the two calves was immunosuppressed by intravenous injection of Dexadreson (10 mL/100 Kg) at days 3, 2 and 1 before infection, the day of infection and the two following days. Infection was obtained by intravenous injection of 2.4 × 10^9^ (immunosuppressed) and 2.5 × 10^9^ iRBC (not immunosuppressed), washed with PBS and resuspended in PBS. The course of infection was followed over a month by regularly monitoring parasite presence in the blood on MGG-stained smears. Cultures were also initiated from the collected red blood cells either in autologous or heterologous conditions using bovine erythrocytes as described previously [13]. All experiments were carried out at Oniris, and adhered to local (Pays de la Loire Ethics Committee) and national guidelines and laws governing experimental work on animals.

### Identification and prevalence of tick species collected on pastures and animals

Ticks were collected on the red deer farm situated in Cádiz from vegetation, using the dragging method throughout the year (10 samplings over the year), and from three different sites representing the principle biotopes (i.e., forest shrub and grasslands) based on the percentage cover of the main grossly classified habitats. Ticks were also collected from animals (red and roe deer on the Cádiz farm). The collected ticks were preserved in 70% ethanol and transported to the laboratory where they were subsequently identified. The aim of this survey was to establish the list of potential vector species for the isolated *Babesia* species in order to select candidate species for subsequent transmission studies.

## Results

### Determination of *Babesia* spp. carrier animals

Hemoparasites could not be detected on any of the Giemsa-stained smears obtained directly from the 112 collected blood samples, indicating that parasitemia in each sample was less than one parasite per 1000 red blood cells (about 10 000 RBC analyzed).

Fifteen cultures (either autologous or heterologous) had to be discarded due to *Trypanosoma* spp. development, despite the use of Amphotericin in the culture medium. Some cultures were *Babesia* positive in autologous red blood cells (26), some in bovine red blood cells (35), and some in both conditions (23). In total, positive cultures were obtained from 37 different red deer. The number of carrier animals differed considerably between the two herds, ranging from 2.9% in the Ciudad Real herd (one isolate) to 46.8% in the Cádiz herd (36 isolates).

### Molecular characterization of the isolates

The molecular characterization of these isolates was carried out by PCR amplification and sequencing of the 18S rRNA gene. The species identity of some isolates (6) that grew initially in both red blood cell types (red deer and cattle), was determined by sequencing 18S rRNA gene from each isolate. This control was performed to confirm that the same parasite was isolated in both culture conditions.

Complete 18S rRNA gene sequences (over 1616 bp length) were obtained for 8 isolates from red deer (232, 262, 265, 279, A2, A20, A21 from the Cádiz herd and the only isolate C32 from the Ciudad Real herd). Four of them were sequenced from the isolate obtained in autologous red blood cells as well as from the isolate obtained by culture in bovine red blood cells. Identical sequences were obtained in each case. Four sequence types (ST) could be distinguished. The first type (ST1) contained four isolates (262, 265, A2 and A21), a second type (ST2) two isolates (279 and C32) and two types (ST3 and ST4) each consisted of one isolate (A20 and 232) (Table 2). Sequence differences between ST1 and the other three sequence types were limited to 1 to 4 nucleotides out of the 1616 bp compared. Blast analysis of these sequences revealed highest identities with *Babesia* sp. Xinjiang [GenBank: DQ159073] (98.94 to 99.40% depending on the red deer sequence type) with 5 to 8 nucleotide changes (Table 2) [25]. Close identities with *Babesia* sp. giraffe i.e., 98.34 to 99.14% identity [GenBank: FJ213677, GenBank:FJ213678 and GenBank: FJ213680] and *Babesia* sp. roan i.e., 98.94 to 99.01% identity [GenBank: FJ213681] were also found [26]. Nucleotide changes between the red deer *Babesia* ST1 sequence and *Babesia* sequences from African ruminants were indeed limited to 5 to 14 nucleotides (Table 2).

The diversity of the parasites isolated from red deer in this farm was further analyzed by obtaining partial 18S rRNA gene sequences covering the 18S rRNA hypervariable region (nt 560 to 935) for 13 more isolates. Most of the isolates (9) had sequences corresponding to sequence types 1 or 2 defined on the basis of the complete 18S rRNA sequence (Table 2). At least two new sequence types (ST5 and ST6) could be defined on the basis of these partial sequences, each of them represented by two isolates. Differences were again limited to one or two nucleotides compared to the main type (ST1).

### Phylogenetic relationships of red deer-infecting *Babesia* isolates

**Figure 1:**
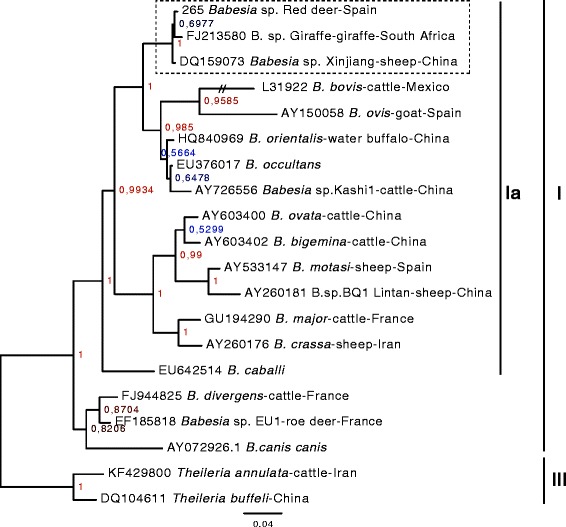
**Consensus tree from the Bayesian analysis of 18 18S rRNA gene sequences of** ***Babesia*** **, with** ***Theileria*** **sequences as the outgroup.** The sequence determined in the present study is highlighted with an arrow, and the monoplyletic group it belongs to by a bold vertical line. The GenBank accession numbers for the retrieved sequences are indicated on the tree. The scale indicates the inferred number of substitutions. The clades as described in previous phylogenetic analyses are indicated [16]. Posterior probabilities are indicated on the nodes. Evolutionary analyses were conducted in MrBayes v3.2.2 [22,23]. The clades I and III from [17] correspond respectively to clades V and VI from [16]. The clade of interest in the present study is framed with a dashed line.

The general 18S rRNA gene sequence alignment used in the first phylogenetic analysis consisted in 20 nucleotide sequences. All positions containing gaps and missing data (sequence extremities) were automatically eliminated in MEGA5. There were a total of 1391 positions in the final data set. Nodes receiving ≥ 95 posterior probability in the Bayesian analysis and/or ≥ 70% bootstrap support in the three other methods were considered statistically supported. The different analyses returned identical topologies: *Babesia* isolated from red deer belonged to the *Babesia* sensu stricto clade (clade VI from [17]), in the subclade Ia [16] (Figure 1 and Additional file 1). The representative of the main sequence type (265) formed a monophyletic group with *Babesia* sp. Xinjiang from sheep in China and *Babesia* sp. Giraffe FJ213580 in South Africa, with strongly supported bootstraps (100% for the three methods) and a Bayesian posterior probability of 1.

**Figure 2:**
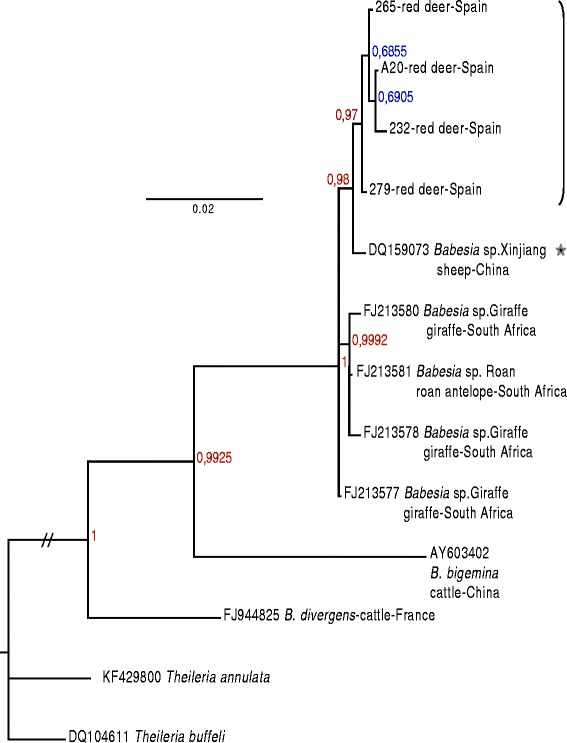
**Consensus tree from the Bayesian analysis of 18S rRNA gene sequences of all complete 18S rRNA gene sequences from the monophyletic group described in Figure** **1** **, with** ***Theileria*** **sequences as the outgroup.** The GenBank accession numbers for the retrieved sequences are indicated on the tree. Sequences were aligned and gaps were removed manually. The Bayesian analysis was conducted in MrBayes v3.2.2 [22,23], and the posterior probabilities are indicated on the nodes. The red deer sequence group is indicated with a bracket, and the closely related sequence of *Babesia* sp. Xinjiang with a star. Long branches have been shortened for presentation purposes and are indicated with //.

Phylogenetic analyses were then performed with the whole set of sequences from this group using a manually optimized alignment, in order to conserve the 17 substitution sites that differentiate the 9 sequences (Table 2). Whatever the method used, the 9 sequences grouped together with strong bootstrap values (100% for the MP and ML, 87% for the NJ) or posterior probability of 1. *Babesia* sequences from red deer grouped together with lower but significant support bootstrap values or posterior probabilities (Figure 2 and Additional file 2). Within this group of 9 sequences, the estimates of evolutionary divergence were extremely low, with a maximum of 0.008 (Additional file 3), but many nodes were not well resolved (several bootstrap values of less than 50%).

### Susceptibility of ruminant erythrocytes to the isolated *Babesia* spp.

**Figure 3:**
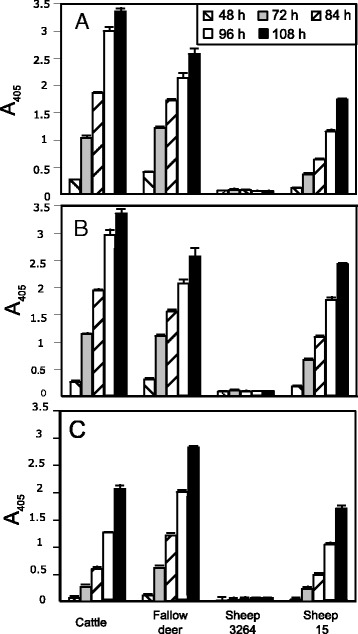
**In vitro susceptibility of cattle, fallow deer and sheep erythrocytes to three** ***Babesia*** **isolates from red deer representing three main 18S rRNA sequence types. A**: isolate 265 (sequence group 1). **B**: isolate 279 (sequence group 2). **C**: isolate 232 (sequence group 4). Growth was monitored by A_405_ measurement of the culture supernatant between 2 and 5 days after culture inoculation.

**Figure 4:**
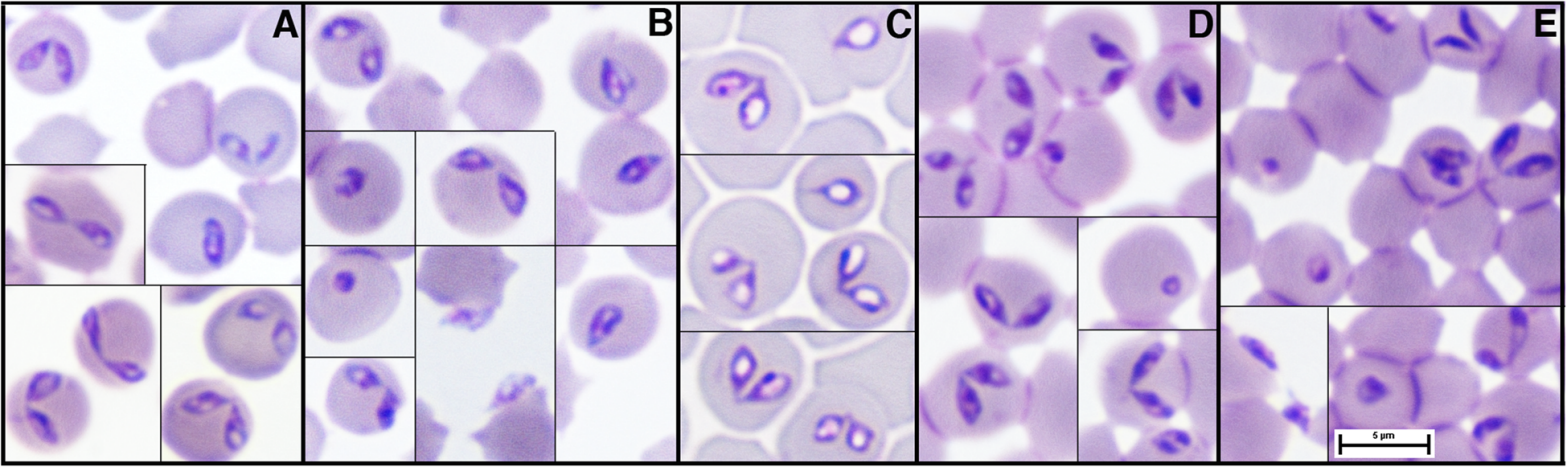
**Morphology of isolate 265 cultivated in different erythrocyte types. A**: red deer erythrocytes. **B**: roe deer erythrocytes. **C**: fallow deer erythrocytes. **D**: cattle erythrocytes. **E**: sheep erythrocytes. In each case, trophozoites and dividing forms are represented. Free merozoites accoled to red blood cells are depicted on panels **B** and **E**. Giemsa stain. Scale bar = 5 μm.

The susceptibilities of bovine, fallow deer and sheep erythrocytes to three isolates (265, 279 and 232) representing three different sequence types were analyzed and compared. Susceptibility was evaluated from the ability of each erythrocyte type to sustain the growth of each isolate. Growth was therefore monitored over a period of 5 days. All three isolates were able to grow in bovine, fallow deer and sheep 15 erythrocytes but unable to grow in sheep 3264 erythrocytes (Figure 3). The growth of isolate 279 was faster in fallow deer erythrocytes than in bovine erythrocytes. The opposite was observed for the other two isolates (Figure 3). Isolate 265 also proved to be able to grow in roe deer erythrocytes (Figure 4).

### Parasite stages observed in vitro and their morphology

Since the parasites were isolated in vitro from asymptomatic red deer, their morphology in vivo could not be described. Indeed, parasites were never detected on smears prepared from the blood of these animals. The morphologies of parasites grown in red deer (autologous), roe deer, fallow deer, cattle and sheep erythrocytes are shown in Figures 4A, B, C, D and E, respectively. The parasites can be classified in the group of large *Babesia*. Whatever the erythrocyte type used to cultivate the isolates, the main forms observed were paired piriforms and single rings. Maltese-cross forms as well as double rings or pairs of parasites were extremely rare.

When cultivated in bovine erythrocytes, the diameters of the ring form ranged from 1.15 μm to 2.09 μm with a mean of 1.62 ± 0.23 μm. The angle between the paired piriforms varied but was usually large, up to 180° (Figure 4A). The size of these dividing parasites ranged from 2.18 to 2.99 × 1.05 to 1.49 μm, with an average size of 2.64 (± 0.21) μm × 1.27 (± 0.13) μm. Free merozoites accolled to the surface of erythrocytes could also be observed (Figures 4B and 4E).

The morphological characteristics of these forms were similar in different eythrocyte types, except in fallow deer erythrocytes, where the plasma was systematically whiter and larger, with a mean width of piriforms increasing to 1.42 ± 0.15 μm.

### Experimental infections in calves

Two intact calves were infected with isolate 265 as representative of the main genetic type present in red deer in Cádiz. Infection was monitored by blood sampling, direct microscopic examination and culture propagation of the parasite from the blood. In the case of the non-immunosuppressed calf, parasites were never observed directly by microscopic examination, but were still present during the first two days post-infection, as confirmed by the positive culture (Table 3). On the contrary, parasites could be detected directly by microscopic examination of blood from the immunosuppressed calf on the day after infection (Day 1), but never at the subsequent time points. Blood cultures were found to be positive up to 28 days post-infection. The increasing delay between culture initiation and parasite detection in the culture indicates that parasitemia in the calf bloodstream decreased with time.

**Table 3:** **In vitro culture of blood collected from immunosuppressed (I) and not immunosuppressed (NI) calves infected with the isolate 265 (Sequence groupe 1)**

**Calf**	**Culture**	**Days post-infection**																				
	**condition**	**1**	**2**	**3**	**4**	**5**	**6**	**7**	**8**	**9**	**10**	**12**	**14**	**16**	**20**	**25**	**28**	**31**	**34**	**36**	**38**	**42**
NI	A	13	12	n	n	n	n	n	n	n	-	n	-	n	n	-	-	n	n	-	-	n
	H	13	12	n	n	n	n	n	n	n	-	n	-	n	n	-	-	n	n	-	-	n
I	A	6	5	12	-	-	23	-	-	-	n	-	n	-	-	n	21	-	-	n	n	-
	H	6	5	12	-	-	18	-	-	-	24	-	n	-	-	n	n	-	-	n	n	-

Cultures were performed in the infected animal’s own red blood cells only (A) and in a mixture with an equal volume of the donor cattle red blood cells (H). n: negative culture, − : blood not sampled at that time point. The number of days at which cultures were found positive is otherwise indicated.

### Identification of tick species collected on pastures and animals

Ticks were collected from vegetation on the red deer farm in Cádiz over a one year period, and the overall density is indicated in Additional file 4. Ticks belonging to 5 genera and 9 different species were identified. *Hyalomma* sp., especially *H. lusitanicum* and *Dermacentor marginatus,* were the most abundant species identified on vegetation (Additional file 4).

Ticks were also collected on deer and identified. Most of the species collected from vegetation were also collected from deer, although *D. marginatus* was unexpectedly absent on animals. In total, 956 ticks were collected from 119 red deer and two main different species of tick were identified: *H. lusitanicum* and *R. annulatus* (Additional file 4)*.*

## Discussion

In the present study, we carried out isolation of *Babesia* sp. by in vitro culture from blood sampled from farmed red deer in central (Ciudad Real, Castilla La Mancha) and southern Spain (Cádiz, Andalucia). Red deer in Europe have been shown to carry parasites belonging to the species *B. capreoli* [27–29] and *B. divergens* [12], in Scotland, Slovenia, Switzerland, Ireland and France, respectively. *Babesia* sp. EU1, present in roe deer, was never identified in red deer present in the same geographical areas [12,28,29]. Parasites of the genus *Babesia* could not be detected in red deer from Northern Spain [10]. In the present study, *B. divergens*, *B. capreoli*, and *Babesia* sp. EU1 were not isolated, despite the use of conditions known to be favorable to their growth from animal blood samples [9,10,13,30]. They share the same tick vector, *I. ricinus* [31–33], and the absence of these three *Babesia* species in the study region may be explained by the absence or very low abundance of this tick species in these regions [34].

However, we report here the isolation and characterization of a new *Babesia* from two geographically distant herds in Spain. The number of red deer carrying this new *Babesia* sp. was found to be very high in the herd from southern Spain, attaining 36 animals on a herd of 77 (46.8%). The abundance of *Babesia* carriers in the other herd was much lower (one isolate, 2.9%). *H. lusitanicum* and *D. marginatus* were the predominant tick species on vegetation in the farm where red deer were highly parasitized by this new *Babesia* species. The identification of ticks collected from the red deer revealed the predominance of *H. lusitanicum.* It therefore represents a prime candidate to perform transmission studies of the isolated *Babesia* and clearly determine its role as a competent vector. However, as tick sampling from deer was performed at only one time point, we cannot exclude a low seasonal activity of the tick vector at the period of deer sampling, and therefore a low abundance on animals. So, if *H. lusitanicum* transmission studies fail, *D. marginatus* could be a good second candidate. The absence of detection of this parasite from deer in Northern Europe is probably due to the absence of its vector in colder areas.

Classification requires that both phenotypic and genotypic methods are applied for sorting out relationships between organisms. Such a polyphasic taxonomy approach has to be generalized, as each method taken alone has its limitations [35]. In our study, we applied a polyphasic taxonomy approach for this new *Babesia*. Phenotypic features such as morphology, erythrocyte as well as host susceptibilities have been studied, to complement the molecular data. Morphology has been a criterium used in the classification of *Babesia* as large or small, but morphology (size, number of merozoite per erythrocytes) is subjected to variations according to the host erythrocyte, the physiological state of the parasite, the in vitro/in vivo state, and also the way smears are performed [36]. However, in our study, morphology was used only as a comparison between isolates grown in the same conditions. Studying the in vivo host range poses serious ethical issues, and some wild species are not amenable to such studies. Therefore, we propose an alternative by testing the in vitro susceptibilities of erythrocytes from different hosts. This approach has been found valuable in the differentiation of *B. capreoli* and *B. divergens* [11], and to relate to the parasite abilities to infect the corresponding host [37]. However, in such tests, the ability of the parasite to establish a long-term culture has to be tested (5 days in the present study), as cell attachment and invasion are not host specific [38,39]. These phenotypic features (morphology, erythrocyte and host susceptibilities) as well as molecular features (18S rRNA gene sequencing) were applied in our study.

The isolated parasites represent a novel *Babesia* species infecting red deer since it is unrelated to any of the previously reported species/isolates in this host. Complete 18S rRNA gene sequences were obtained for 8 isolates from the two herds. All sequences were closely related, with identities ranging from 99.46 to 99.94%. Species delineation on the basis of sequence identities is controversial, and there is no rule about the level of genetic difference on a particular gene that defines a species. In their study of the phylogeny of *Babesia* from sheep and goat, Schnittger et al. [40] estimated that, within the genus *Babesia*, isolates with an 18S rRNA gene identity higher than 97.6% should be considered as belonging to the same species. In the same study, isolates were considered as belonging to separate species when this identity was less than 96.6%. So, based on the 18S rRNA molecular marker, the characterized isolates belong to the same species. Biological characteristics also support that these isolates belong to the same species. Despite the considerable and unusual genetic variability observed at such a small geographic scale, the morphology of the 37 isolates as assessed by in vitro cultivation was similar. Altogether six 18S rRNA gene sequence types (4 and 2 sequence types of complete and partial sequence analysis respectively) could be identified in the red deer isolates. An additional common feature of three isolates as defined by different representative sequence types was that a similar in vitro susceptibility was observed when cultivated in cattle, sheep and fallow deer erythrocytes. All isolates grew in cattle as well as in fallow deer erythrocytes. However, all three representative isolates were refractory to growth in one of two tested sheep erythrocytes while they grew well in the other. Such growth variation in sheep erythrocytes seems to be a common feature and has been observed with other *Babesia* species, namely *B. divergens* [37] and another sheep pathogen from China, *Babesia* sp. BQ1 [30]. From the complementary molecular and biological features described from several red deer isolates, we can conclude that despite their genetic variation, they belong to the same new species.

Phylogenetic analysis placed this new species in the *Babesia* sensu stricto clade VI [17], as well as in the clade Ia described by Lack et al. [16]. Whatever the method used to build the tree (Neighbor-Joining, Parsimony, Maximum Likelihood, Bayesian), this *Babesia* species from red deer formed a monophyletic group that comprises *Babesia* sp. Xinjiang from China and sequences of *Babesia* from giraffe in South Africa with highly significant bootstrap support (100%) or a Bayesian posterior probability of 1. Recent phylogenetic studies based on the Bayesian [17] and/or maximum parsimony analysis [16] of 18S rRNA gene sequences from Piroplasmida also placed the 18S rRNA gene sequences of *Babesia* infecting sheep, giraffe and roan antelope in the same monophyletic group with high statistical support.

It has been previously suggested that the *Babesia* sp. identified in Africa on giraffe and roan antelope as well as *Babesia* sp. Xinjiang from sheep in China are variants of the same species [26]. In the present study, the identities of the 18S rRNA gene sequences of the members of this group were all higher than 96.6% (over 98.3%), and they could thus be considered as variants of the same species according to [40]. However, we strongly believe that the assignment of an isolate to a species on the basis of sequence data must also be correlated with biological characteristics. For example, *B. divergens* and *B. capreoli* are two different species, even if the identity based on 18S rRNA is higher than 99%, because they have distinct biological features, in vitro (erythrocyte susceptibility tests) as well as in vivo, namely the ability to infect cattle, gerbils (*Meriones unguiculatus*) and humans [11]. In the present case, biological features cannot be compared for the South African parasites, since only molecular data are available [26]. These data were described for the *Babesia* sp. Xinjiang isolate [41], and none of the biological data that could be compared between the *Babesia* sp. Xinjiang isolate and the red deer isolates highlighted a difference between them:

1. They are morphologically comparable.
2. For both, in vitro ability to infect sheep and cattle erythrocytes has been demonstrated using the same culture conditions, even if their host of origin is either sheep or red deer. *Babesia* sp. Xinjiang is also able to develop in vitro in cervid erythrocytes (namely those of sika deer, *Cervus nippon*).
3. For both, the inability to establish a persistent infection in experimentally infected calves has also been demonstrated. The experimental infection of intact calves performed in our study demonstrated that the red deer parasites were only able to persist for a short period in the blood of infected animals subjected to an immunosuppressive treatment, with a rapid decrease in parasitemia. A similar experiment was conducted with the *Babesia* sp. Xinjiang isolate, on splenectomized calves [37]. As in our experiment, parasites could not be visualized on blood smears prepared after infection, nor detected by PCR.

The segregation of analyzed 18S rRNA gene sequence types of the isolate into one single clade, the common morphology of the in vitro cultured parasite, and the same susceptibility patterns of erythrocytes in cattle, sheep and fallow deer strongly support that the red deer *Babesia* species and the isolate *Babesia* sp. Xinjiang represent a single species. However, naming *Babesia* identified from different hosts in different continents as the same species poses the problem of geographical isolation, as a species is defined as a population of individuals capable of interbreeding, but these isolates are reproductively isolated as they stem from different continents.

Therefore, we propose the name *Babesia pecorum* as a newly characterized species isolated from red deer in Spain. The species name issued from the Pecora infraorder of the described host, with the plural genitive case *pecorum*: “of the Pecora”. Members of the monophyletic group which includes *Babesia* sp. Xinjiang from China [25], and the *Babesia* from South African ruminants [26] could be added later as members of this newly described species, since this infraorder groups their host families i.e. bovidae, giraffidae and cervidae.

Even if the molecular and biological data strongly suggest their belonging to the same species, additional data are necessary to surely gather isolates from different continents described on different hosts into the same species. An essential step would be to demonstrate either that *B. pecorum* can infect sheep, or *Babesia* sp. Xinjiang can infect red deer, or at least cervids present in the same geographical areas as the sheep parasite. Such data from the South African parasites would have to wait these parasites to be isolated in order to perform such studies. Up to now, only molecular data are available for these parasites. Information about the vector may not be as relevant to decide about the species status of the transmitted parasite, since different ticks can transmit the same *Babesia* species (i.e. *B. bigemina*).

### Taxonomic review

Family: Babesiidae.

Parasite: Babesia pecorum sp. nov.

Type host: Red deer (*Cervus elaphus*).

Type locality: Spain.

Vector: unknown (*Hyalomma lusitanicum* suspected).

Etymology: The species name issued from the Pecora infraorder of the described host, with the plural genitive case *pecorum*: «of the Pecora».

Description: ring form (1.15 μm to 2.09 μm with a mean of 1.62 ± 0.23 μm); dividing parasites (2.18 to 2.99 × 1.05 to 1.49 μm, with an average size of 2.64 (± 0.21) μm × 1.27 (± 0.13) μm).

Type material: stained thin blood smears of the Spanish (Cádiz) isolate 265 from red deer blood (ZS-133), genomic DNA extracted from isolate 265 (ZS132) have been deposited at the Protist collection of the National Natural History of Paris [42]. The holotype 265 is being maintained as cryostabilates in the same collection under the reference ZS-131. All the other syntypes described in this paper are maintained at Oniris as cryostabilates.

## Additional files

Additional file 1:
**Complementary phylogenetic analyses of the complete 18S rRNA sequences used in Figure** 1 **.** The GenBank accession numbers for the retrieved sequences are indicated on the tree. The analysis involved 20 nucleotide sequences that were aligned and all positions containing gaps and missing data were eliminated manually. There were a total of 1391 positions in the final dataset. Evolutionary analyses were conducted in MEGA5 [18]. **(A)** Neighbor-Joining method. The bootstrap consensus tree inferred from 500 replicates is taken to represent the evolutionary history of the taxa analyzed. Branches corresponding to partitions reproduced in less than 50% bootstrap replicates are collapsed. The percentage of replicate trees in which the associated taxa clustered together in the bootstrap test (500 replicates) are shown next to the branches. The evolutionary distances were computed using the Tamura 3-parameter method and are in the units of the number of base substitutions per site. The rate variation among sites was modeled with a gamma distribution (shape parameter = 0.4). **(B)** Maximum Parsimony method. The bootstrap consensus tree inferred from 500 replicates is taken to represent the evolutionary history of the taxa analyzed. Branches corresponding to partitions reproduced in less than 50% bootstrap replicates are collapsed. The percentage of replicate trees in which the associated taxa clustered together in the bootstrap test (500 replicates) are shown next to the branches. The MP tree was obtained using the Subtree-Pruning-Regrafting (SPR) algorithm with search level 1 in which the initial trees were obtained by the random addition of sequences. **(C)** Maximum Likelihood method based on the Tamura 3-parameter model. The bootstrap consensus tree inferred from 500 replicates is taken to represent the evolutionary history of the taxa analyzed. Branches corresponding to partitions reproduced in less than 50% bootstrap replicates are collapsed. The percentage of replicate trees in which the associated taxa clustered together in the bootstrap test (500 replicates) are shown next to the branches. Initial tree(s) for the heuristic search were obtained automatically by applying Neighbor-Join and BioNJ algorithms to a matrix of pairwise distances estimated using the Maximum Composite Likelihood (MCL) approach, and then selecting the topology with superior log likelihood value. A discrete Gamma distribution was used to model evolutionary rate differences among sites (5 categories (+*G*, parameter = 0.1647)). The bootstrap support values are indicated on the nodes. Additional file 2:
**Complementary phylogenetic analyses of the complete 18S rRNA sequences used in Figure** 2 **.** The GenBank accession numbers for the retrieved sequences are indicated on the tree. The analysis involved 13 nucleotide sequences that were aligned. All positions containing gaps and missing data were eliminated. There were a total of 1461 positions in the final dataset. Evolutionary analyses were conducted in MEGA5 [18]. **(A)** Neighbor-Joining method. The bootstrap consensus tree inferred from 1000 replicates is taken to represent the evolutionary history of the taxa analyzed. Branches corresponding to partitions reproduced in less than 50% bootstrap replicates are collapsed. The percentage of replicate trees in which the associated taxa clustered together in the bootstrap test (1000 replicates) are shown next to the branches. The evolutionary distances were computed using the Tamura 3-parameter method and are in the units of the number of base substitutions per site. The rate variation among sites was modeled with a gamma distribution (shape parameter = 0.05). **(B)** Maximum Parsimony method. The bootstrap consensus tree inferred from 1000 replicates is taken to represent the evolutionary history of the taxa analyzed. Branches corresponding to partitions reproduced in less than 50% bootstrap replicates are collapsed. The percentage of replicate trees in which the associated taxa clustered together in the bootstrap test (1000 replicates) is shown next to the branches. The MP tree was obtained using the Subtree-Pruning-Regrafting (SPR) algorithm with search level 1 in which the initial trees were obtained by the random addition of sequences (10 replicates). **(C)** Maximum Likelihood method based on the Tamura 3-parameter model. The bootstrap consensus tree inferred from 1000 replicates is taken to represent the evolutionary history of the taxa analyzed. Branches corresponding to partitions reproduced in less than 50% bootstrap replicates are collapsed. The percentage of replicate trees in which the associated taxa clustered together in the bootstrap test (1000 replicates) is shown next to the branches. Initial tree(s) for the heuristic search were obtained automatically by applying Neighbor-Join and BioNJ algorithms to a matrix of pairwise distances estimated using the Maximum Composite Likelihood (MCL) approach, and then selecting the topology with superior log likelihood value. A discrete Gamma distribution was used to model evolutionary rate differences among sites (5 categories (+*G*, parameter = 0.1583)). Additional file 3:
**Estimates of evolutionary divergence between sequences.** The number of base substitutions per site from between sequences are shown. Analyses were conducted using the Tamura3-parameter model. The rate variation among sites was modeled with a gamma distribution (shape parameter = 0.05). All positions containing gaps and missing data were eliminated manually. There were a total of 1,461 positions in the final dataset. Additional file 4:
**Identification and density of collected ticks (ticks/hectare).** Ticks were collected on the vegetation on the Cádiz deer farm throughout a monthly survey during one annual cycle, and on red deer.
