# Supplementary material for: Isolation and characterization of Babesia pecorum sp. nov. from farmed red deer (Cervus elaphus)
Source: Vet Res. 2014 Aug 26;45(1):78. doi: 10.1186/s13567-014-0078-7 (PMC4158131; doi:10.1186/s13567-014-0078-7)
Supplement: Additional file 2: — Complementary phylogenetic analyses of the complete 18S rRNA sequences used in Figure 2 . The GenBank accession numbers for the retrieved sequences are indicated on the tree. The analysis involved 13 nucleotide sequences that were aligned. All positions containing gaps and missing data were eliminated. There were a total of 1461 positions in the final dataset. Evolutionary analyses were conducted in MEGA5 [18]. (A) Neighbor-Joining method. The bootstrap consensus tree inferred from 1000 replicates is taken to represent the evolutionary history of the taxa analyzed. Branches corresponding to partitions reproduced in less than 50% bootstrap replicates are collapsed. The percentage of replicate trees in which the associated taxa clustered together in the bootstrap test (1000 replicates) are shown next to the branches. The evolutionary distances were computed using the Tamura 3-parameter method and are in the units of the number of base substitutions per site. The rate variation among sites was modeled with a gamma distribution (shape parameter = 0.05). (B) Maximum Parsimony method. The bootstrap consensus tree inferred from 1000 replicates is taken to represent the evolutionary history of the taxa analyzed. Branches corresponding to partitions reproduced in less than 50% bootstrap replicates are collapsed. The percentage of replicate trees in which the associated taxa clustered together in the bootstrap test (1000 replicates) is shown next to the branches. The MP tree was obtained using the Subtree-Pruning-Regrafting (SPR) algorithm with search level 1 in which the initial trees were obtained by the random addition of sequences (10 replicates). (C) Maximum Likelihood method based on the Tamura 3-parameter model. The bootstrap consensus tree inferred from 1000 replicates is taken to represent the evolutionary history of the taxa analyzed. Branches corresponding to partitions reproduced in less than 50% bootstrap replicates are collapsed. The percentage of replicate tree [file 13567_2014_78_MOESM2_ESM.doc]

**A.**

**B.**

**C.**
