# Supplementary material for: Isolation and characterization of Babesia pecorum sp. nov. from farmed red deer (Cervus elaphus)
Source: Vet Res. 2014 Aug 26;45(1):78. doi: 10.1186/s13567-014-0078-7 (PMC4158131; doi:10.1186/s13567-014-0078-7)
Supplement: Additional file 3: — Estimates of evolutionary divergence between sequences. The number of base substitutions per site from between sequences are shown. Analyses were conducted using the Tamura3-parameter model. The rate variation among sites was modeled with a gamma distribution (shape parameter = 0.05). All positions containing gaps and missing data were eliminated manually. There were a total of 1,461 positions in the final dataset. [file 13567_2014_78_MOESM3_ESM.doc]

|  | 1 | 2 | 3 | 4 | 5 | 6 | 7 | 8 | 9 | 10 | 11 | 12 | 13 |
| --- | --- | --- | --- | --- | --- | --- | --- | --- | --- | --- | --- | --- | --- |
| 1- *T. buffeli* |  |  |  |  |  |  |  |  |  |  |  |  |  |
| 2- *T. annulata* | 0.043 |  |  |  |  |  |  |  |  |  |  |  |  |
| 3- *B. divergens* | 0.293 | 0.339 |  |  |  |  |  |  |  |  |  |  |  |
| 4- *B. bigemina* | 0.432 | 0.532 | 0.144 |  |  |  |  |  |  |  |  |  |  |
| 5- *B*. sp. Xinjiang | 0.397 | 0.491 | 0.108 | 0.115 |  |  |  |  |  |  |  |  |  |
| 6- *B*. sp. Giraffe FJ213580 | 0.438 | 0.507 | 0.108 | 0.125 | 0.007 |  |  |  |  |  |  |  |  |
| 7- *B*. sp. Giraffe FJ213578 | 0.425 | 0.507 | 0.111 | 0.129 | 0.007 | 0.007 |  |  |  |  |  |  |  |
| 8- *B*. sp. Giraffe FJ213577 | 0.390 | 0.450 | 0.096 | 0.111 | 0.004 | 0.003 | 0.004 |  |  |  |  |  |  |
| 9- *B*. sp. Roan FJ213581 | 0.413 | 0.478 | 0.102 | 0.118 | 0.005 | 0.001 | 0.005 | 0.001 |  |  |  |  |  |
| 10- 265 | 0.401 | 0.468 | 0.107 | 0.111 | 0.004 | 0.007 | 0.007 | 0.004 | 0.005 |  |  |  |  |
| 11- A20 | 0.389 | 0.468 | 0.107 | 0.111 | 0.003 | 0.007 | 0.007 | 0.004 | 0.005 | 0.001 |  |  |  |
| 12- 279 | 0.409 | 0.477 | 0.105 | 0.109 | 0.003 | 0.006 | 0.006 | 0.003 | 0.004 | 0.001 | 0.001 |  |  |
| 13- 232 | 0.409 | 0.491 | 0.113 | 0.118 | 0.004 | 0.008 | 0.008 | 0.005 | 0.007 | 0.002 | 0.001 | 0.003 |  |
