# Supplementary material for: Isolation and characterization of Babesia pecorum sp. nov. from farmed red deer (Cervus elaphus)
Source: Vet Res. 2014 Aug 26;45(1):78. doi: 10.1186/s13567-014-0078-7 (PMC4158131; doi:10.1186/s13567-014-0078-7)
Supplement: Additional file 4: — Identification and density of collected ticks (ticks/hectare). Ticks were collected on the vegetation on the Cádiz deer farm throughout a monthly survey during one annual cycle, and on red deer. [file 13567_2014_78_MOESM4_ESM.doc]

| Tick identification | Stage^1^ | Average density | Percentage of the collected ticks on red deer |
| --- | --- | --- | --- |
| *H. lusitanicum* | A | 96.5 | 58 |
| *H. excavatum* | A | 1.1 |  |
| *Hyalomma* spp. | A | 2.6 |  |
|  | N | 846.8 |  |
|  | L | 1653 |  |
| *R. bursa* | A | 11.1 | 0.1 |
| *R. pusillus* | A | 5.4 | 3.7 |
|  | N | 26.9 |  |
| *R. annulatus* | A | 2.7 | 36 |
| *Rhipicephalus* spp. | L | 0.2 |  |
| *D. marginatus* | A | 56.7 |  |
|  | L | 1500.8 |  |
| *I. ricinus* | A | 0.7 |  |
| *I. ventalloi* | A | 0.7 |  |
|  | N | 3.6 |  |
|  | L | 0.2 |  |
| *Ixodes* spp. | L | 2.7 |  |
| *H. punctata* | A | 0.9 |  |
|  | N | 22.5 |  |
| Not identified |  |  | 2.2 |

^1^ Stage: A=Adult, N=nymph, L=larvae.
